# Supplementary figures and images for: Cdhr1a and pcdh15b may link photoreceptor outer segments with calyceal processes revealing a potential mechanism for cone-rod dystrophy
Source: eLife. 2026 Apr 17;13:RP102258. doi: 10.7554/eLife.102258 (PMC13090022; doi:10.7554/eLife.102258)

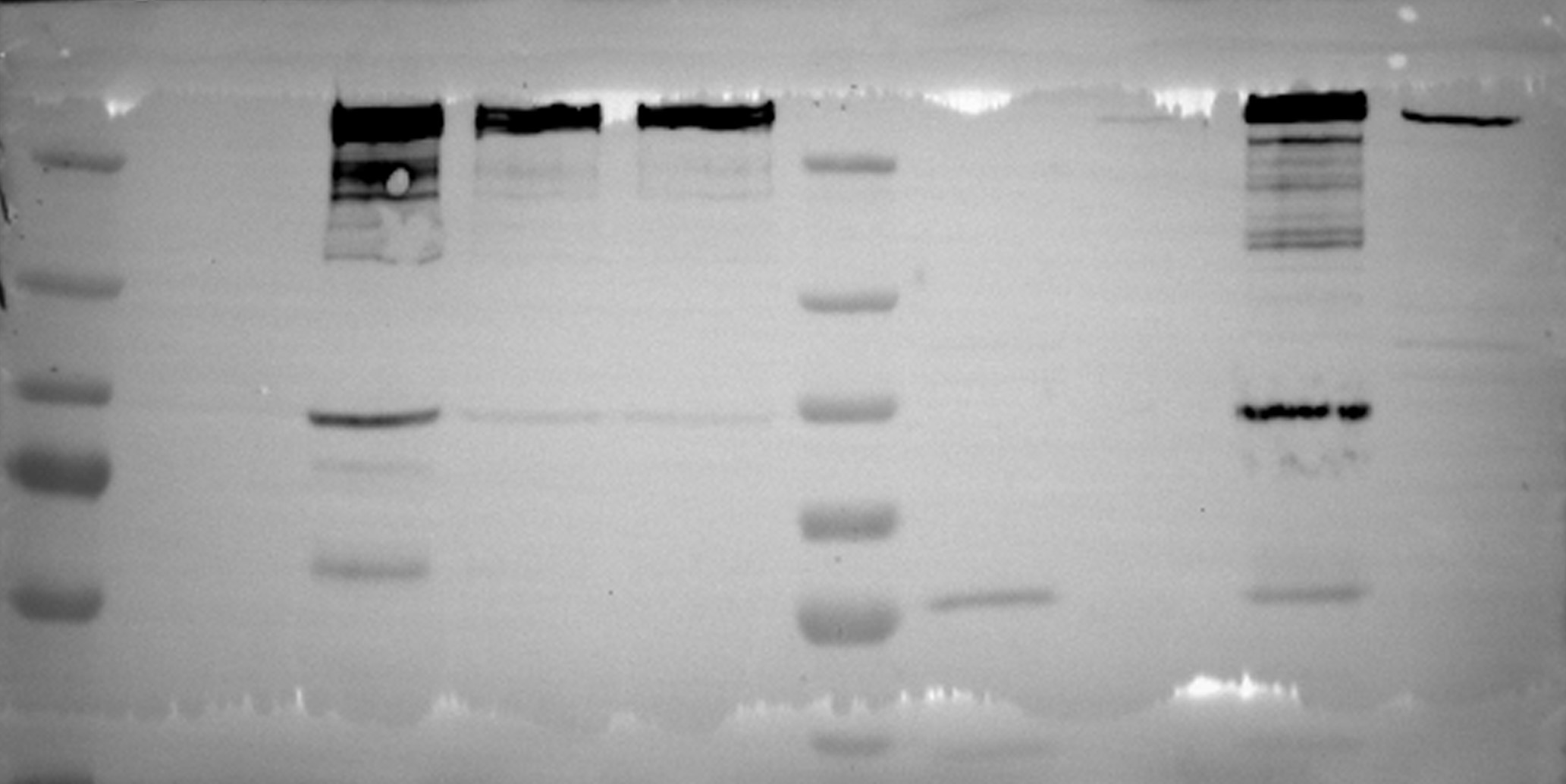

Supplement: Figure 3—source data 1. [file elife-102258-fig3-data1.zip › Figure 3 source data 1/Figure 3 panel A MYC blot.jpg]

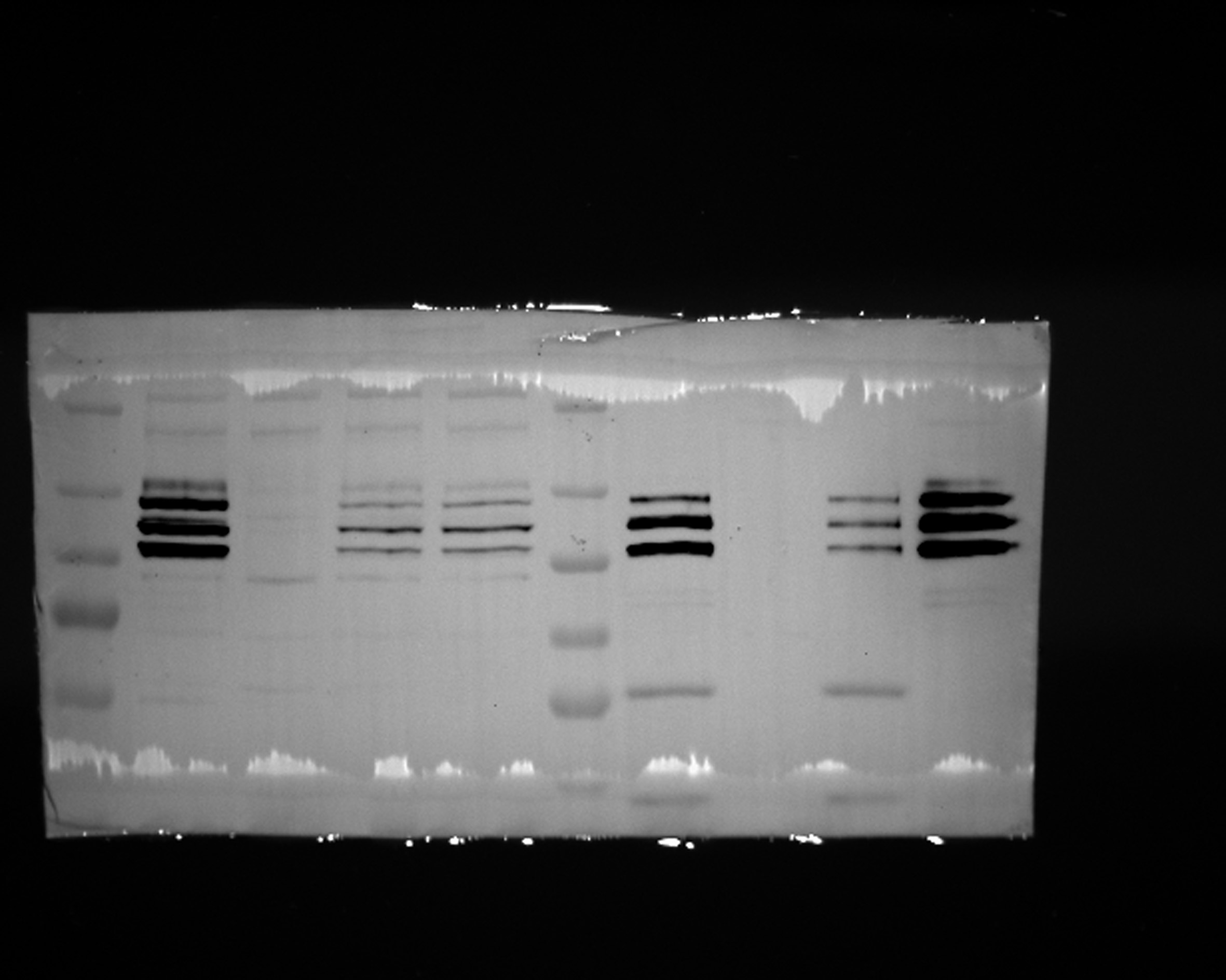

Supplement: Figure 3—source data 1. [file elife-102258-fig3-data1.zip › Figure 3 source data 1/Figure 3 panel A FLAG blot.jpg]
