# Supplementary material for: Cdhr1a and pcdh15b may link photoreceptor outer segments with calyceal processes revealing a potential mechanism for cone-rod dystrophy
Source: eLife. 2026 Apr 17;13:RP102258. doi: 10.7554/eLife.102258 (PMC13090022; doi:10.7554/eLife.102258)
Supplement: Figure 3—source data 2. [file elife-102258-fig3-data2.zip › Figure 3 source data 2/WB source material figure.pdf]

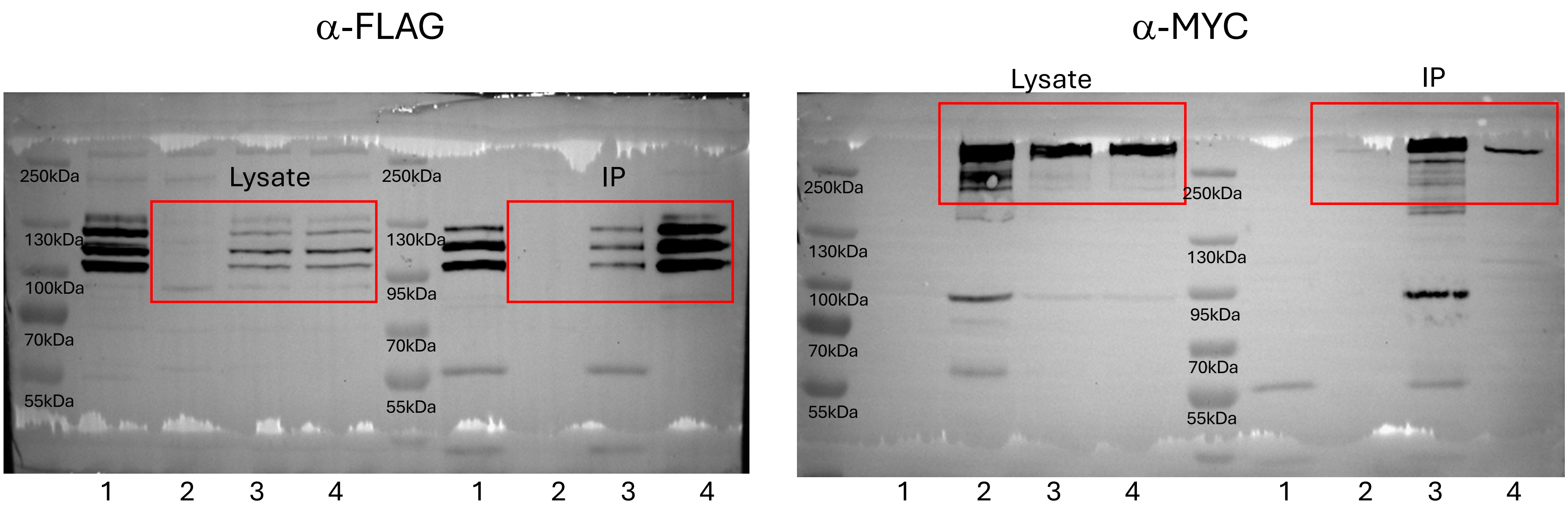

1: cdhr1a-FLAG
2: pcdh15b-MYC (IP-FLAG)
3: cdhr1a-FLAG + pcdh15b-MYC (IP-MYC)
4: cdhr1a-FLAG + pcdh15b-MYC (IP-FLAG)
